# Genomic insight into *Aquimarina longa* SW024^T^: its ultra-oligotrophic adapting mechanisms and biogeochemical functions

**DOI:** 10.1186/s12864-015-2005-3

**Published:** 2015-10-12

**Authors:** Tingting Xu, Min Yu, Heyu Lin, Zenghu Zhang, Jiwen Liu, Xiao-Hua Zhang

**Affiliations:** College of Marine Life Sciences, Ocean University of China, 5 Yushan Road, Qingdao, 266003 P. R. China; Institute of Evolution & Marine Biodiversity, Ocean University of China, Qingdao, 266003 China

**Keywords:** *Aquimarina longa*, Genome analysis, Genome comparison, Oligotrophic bacterium

## Abstract

**Background:**

South Pacific Gyre (SPG) is the largest and clearest gyre in the world, where the concentration of surface chlorophyll *a* and primary production are extremely low. *Aquimarina longa* SW024^T^ was isolated from surface water of the SPG center. To understand how this bacterium could survive in this ultra-oligotrophic oceanic environment and its function in biogeochemical cycle, we sequenced the genome of *A. longa* SW024^T^ and performed extensive genomic analyses.

**Methods:**

Genomic DNA was extracted and sequenced using Illumina Hiseq 2000 and Miseq platform. Genome annotation, genomic comparison and phylogenetic analyses were performed with the use of multiple bioinformatics tools like: BLAST+ 2.2.24, Glimmer3.0, RAST server, Geneious 4.8.5, ClustalW2 and MEGA5. Physiological and morphological features were tested by bacterial culture, electron microscopy, fluorescence microscopy and exopolysaccharides extraction.

**Results:**

Analysis of seven *Aquimarina* genomes and 30 other genomes of *Flavobacteriaceae* isolated from seawater showed that most of the strains had low DNA G + C contents, and *Aquimarina* had larger genomes than other strains. Genome comparison showed varying genomic properties among seven *Aquimarina* genomes, including genome sizes and gene contents, which may warrant their specific adaptive strategies. Genome of *A. longa* SW024^T^ was further compared with the genomes of two other *Aquimarina* species which were also isolated from the SPG and *A. longa* SW024^T^ appeared to have much more genes related to replication, recombination and repair. As a copiotroph, *A. longa* SW024^T^ is long in length, and possesses large genome size and diverse transporters. However, it has also evolved many properties to survive in the oligotrophic marine environment. This bacterium grew better on solid medium than in liquid medium, suggesting it may be liable to attach to particle surfaces in order to survive in the nutrient-limiting environment. Gliding motility and the capacity to degrade various polymers possibly allow the bacterium to grow on detritus particles and use polymeric substances as carbon and energy sources. Moreover, genes related to carbon, nitrogen, and sulfur metabolisms were identified, which showed that *A. longa* SW024^T^ might be involved in various elemental cycles.

**Conclusions:**

Genomic comparison of *Aquimarina* genus exhibits comprehensive capabilities of the strains to adapt to diverse marine environments. The genomic characteristics of *A. longa* SW024^T^ reveal that it evolves various strategies to cope with both copiotrophic and ultra-oligotrophic marine environment, which provides a better understanding of the survival abilities of bacteria in prevalent and even extreme oceanic environments. Furthermore, carbon, nitrogen and sulfur utilization of *A. longa* SW024^T^ may represent its potential functions in the global biogeochemical cycle.

**Electronic supplementary material:**

The online version of this article (doi:10.1186/s12864-015-2005-3) contains supplementary material, which is available to authorized users.

## Background

Among the major taxa of marine bacterioplankton, members of the *Bacteroidetes* are frequently found enriched on organic matter particles and are specialists for degrading high molecular weight compounds of both dissolved and particulate marine organic matters, implying a major role they play in the marine carbon cycle [1, 2]. *Bacteroidetes* have been shown to comprise the largest fraction of bacteria consuming chitin, polysaccharides and proteins, but the smallest fraction consuming amino acids [3]. Luo [4] found that *Bacteroidetes* clades had a greater fraction of genes encoding periplasmic proteins and a lower fraction of genes encoding inner membrane proteins in their metatranscriptomes than in their genomes and metagenomes, corroborating the macromolecule degradation process requiring cell surface associated or extracellular hydrolases. Some representatives of the *Bacteroidetes* phylum such as *Flavobacteriaceae* were frequently found attached to aggregates and appeared during an algae-bloom collapse [5]. They were also known to move over surfaces by gliding motility. The genus *Aquimarina* is a member of the family *Flavobacteriaceae* [6] and was first described in 2005 [7]. Up to now, a total of 18 species in the genus *Aquimarina* have been recognized, and all of them were isolated from marine environments.

South Pacific Gyre (SPG) is the largest gyre in the world, which has the lowest surface chlorophyll *a* (Chl *a*) concentration [8] and is believed to be the clearest water in the world [9]. Comparing with the gyre edge, the low concentration of Chl *a*, ammonium, nitrate and phosphate in the gyre center makes the region an ultra-oligotrophic oceanic environment [8]. *Aquimarina longa* SW024^T^ was a new species isolated from the surface water of a station (U1367) located in the central gyre of SPG [10, 11]. It is long-rod in shape, 0.3 μm in width and 3.0–66.0 μm in length, non-flagellated and motile by gliding. Colonies on marine agar 2216 (MA; Becton Dickinson) are yellow, producing pigment with maximum absorption at 453 nm and 479 nm. Some substrates can be hydrolyzed by this bacterium, including chitin, gelatin, DNA, and Tweens 20, 40 and 80. It is also resistant to many antibiotics, such as benzylpenicillin, carbenicillin, cefuroxime, cephalosporin V, polymyxin B, gentamicin, kanamycin, neomycin, tetracycline, cefoperazone and streptomycin. The specific cellular morphology and the physiological function of this bacterium may provide some advantages for its survival in the ultra-oligotrophic environment.

Nowadays, more and more bacterial genomes have been sequenced and analyzed, however, the genomes of genus *Aquimarina* which is closely associated with marine environment have not been analyzed systematically, except for *A. agarilytica* ZC1^T^, which was isolated from marine red alga and had been proved to have agarolytic activity [12]. In this context, the present study aims to provide a better understanding of the survival mechanisms and biogeochemical role of *A. longa* SW024^T^ in the ultra-oligotrophic environment by extensive genomic analyses. In addition, comparison with other publicly available genome sequences from members of *Aquimarina* reveals that they are diverse in their genome sizes and gene contents, which might warrant their specific adaptive strategies.

## Methods

### Bacterial growth and DNA extraction

*A. longa* SW024^T^ was routinely grown aerobically in marine broth 2216 (MB; BD) or on MA at 28°C. A series of dilutions (1:2, 1:5, 1:10, 1:20 and 1:50) of MB and MA media were used to determine its growth in nutrient-limiting conditions. After being inoculated in MB or streaked on MA, the bacteria were cultured at 28°C for two weeks. Genomic DNA was extracted from the cells by using phenol-chloroform-isoamylic alcohol extraction protocol described by Marmur [13], and the 16S rRNA genes were sequenced to validate the obtained strains.

### Genome sequencing, analysis and annotation

The genome of *A. longa* SW024^T^ was sequenced using the Illumina Hiseq 2000 with 2 kb and 3 kb mate-pair libraries and Illumina Miseq with a 400 bp paired-end library, achieving about 241-fold coverage. The reads were assembled using GS *de novo* assembler software.Putative genes were identified using GLIMMER 3.0 [14]. Annotation was performed with BLAST+ 2.2.24 [15] searching against databases, including the National Center for Biotechnology Information (NCBI) non-redundant proteins (NR) [16], Clusters of Orthologous Groups of proteins (COG) [17], Kyoto encyclopedia of genes and genomes (KEGG) [18] and Gene ontology (GO) [19]. The criteria used to assign function to a protein translated by predicted open reading frames (ORFs) were a minimum cut-off of 30% identity and at least three best hits among the NR, COG, KEGG and GO databases.

### Genomic comparison

The genomes of *A. pacifica* SW150^T^, *A. megaterium* XH134^T^ and *A. macrocephali* JAMB N27^T^ were previously sequenced in the lab, in which the first two strains were also isolated from SPG [20, 21] (Table 1). The genome sequences of *A. latercula* DSM 2041^T^, *A. muelleri* DSM 19832^T^, *A. agarilytica* ZC1^T^ and 30 other *Flavobacteriaceae* strains were obtained from NCBI (Table 1, Additional file 1). The ORFs of all these genomes were predicted conformably using RAST server [22] and translated to amino acid by Geneious 4.8.5 [23]. Orthologous proteins were defined as reciprocal best hit proteins with a minimum 50% identity and 70% of the length of the query protein [24], calculated by the BLAST algorithm. Proteins existed in all genomes subsequently were aligned using ClustalW2 [25], the resulting alignments were concatenated to provide the whole-genome alignment. Phylogenetic analysis was performed by using the MEGA5 software package [26] and the neighbor-joining tree [27] was constructed and validated with 1000 bootstraps. Genomic features and function annotation of predicted proteins from *A. longa* SW024^T^ were first compared with those of the six genomes from the same genus and then further compared with the two strains isolated from SPG, using BLASP with an E-value cut-off of 1e-5. Pan-genome and orthologous cluster analyses were performed with pan-genome analysis pipeline [28].

### Cellular morphology and chitinase activity

*A. longa* SW024^T^ was cultured in MB for one day, and then observed with a transmission electron microscope (TEM-1200EX, JEOL, Tokyo, Japan). It was also stained with DAPI and viewed by fluorescence microscopy with a ×100 oil immersion lens (Nikon Eclipse 50i, Japan). The intracellular structure was shown using ultramicrotomy and observed with transmission electron microscope. Chitinase activity was observed using chitin agar following the method described by Hsu and Lockwood [29].

### Exopolysaccharides (EPS) analyses

EPS extraction and analyses were followed the method described by Balsanelli et al. [30] with some modification. Briefly, *A. longa* SW024^T^ was grown in MB medium at 28°C and 170 rpm. After 3 days, 10 ml of the bacterial cultures were centrifuged and the supernatant were precipitated with 3 volumes of cold ethanol for 24 hours at 4°C and centrifuged for 10 minutes at 4°C and 8,000 g. The precipitate was dissolved in deionized water and dialyzed against MilliQ water. The dialyzed sample was lyophilized and resuspended in 1 ml of distilled water. Total sugar concentration of the samples was determined with phenol/sulfuric acid [31], using glucose as standard. Three independent experiments were performed and the mean concentration was calculated.

### Nucleotide sequence accession numbers

The genome project has been deposited in the Genome On Line Database (GOLD) under the accession number Gi0050938. This Whole Genome Shotgun project has been deposited at DDBJ/EMBL/GenBank under the accession number AVQK00000000. The version described in this paper is version AVQK01000000.

## Results and discussion

### Genome features of *A. longa* SW024^T^ and other *Aquimarina* bacteria

The genome of *A. longa* SW024^T^ was composed of 5,506,799 bp, and the calculated G + C content was 31.45 %. A total of 90 contigs ranging from 616 bp to 487,322 bp (the N50 and N90 contig sizes were 288,357 bp and 66,130 bp, respectively) were obtained and combined into 67 scaffolds ranging from 1130 bp to 837,171 bp (the N50 and N90 scaffold sizes were 309,215 bp and 66,130 bp, respectively). A total of 4822 ORFs were identified within the *A. longa* SW024^T^ genome (Table 1). Among the predicted genes, 2507 (51.99 %) were found in COG categories, 1455 (30.17 %), 4168 (86.44 %) and 1939 (40.21 %) genes were applicable within the KEGG, NR and GO databases, respectively.

**Table 1:** Summary of genomic information of seven *Aquimarina* genomes

Strains	Size (Mb)	G + C content (%)	Contig No.	ORF No.	Orthologous cluster No.	Specific genes	GenBank accession No.	Isolation environment
*A. longa* SW024^T^	5.50	31.45	90	4822	4521	1522	AVQK00000000	Surface seawater of SPG
*A. pacifica* SW150^T^	5.26	33.49	145	4368	4230	1280	JACC00000000	Surface seawater of SPG
*A. megaterium* XH134^T^	6.21	32.93	170	5425	5206	1039	JACB00000000	Surface seawater of SPG
*A. macrocephali* JAMB N27^T^	6.06	32.93	169	5414	5211	1088	JACA00000000	Marine sediment off Kagoshima, Japan
*A. latercula* DSM 2041^T^	6.24	32.21	31	5522	5165	1831	AUMK00000000	Outflow of a marine aquarium in La Jolla, California, USA
*A. muelleri* DSM 19832^T^	4.90	31.33	106	4085	3942	869	AUML00000000	Seawater of Amursky Bay, Sea of Japan
*A. agarilytica* ZC1^T^	4.25	32.81	131	3571	3451	1711	AHHE00000000	Surface of marine red alga, collected near Nan Ao Island, Guangdong province, China

General information of seven *Aquimarina* genomes used for comparison and analysis was summarized in Table 1. The contig numbers of the draft genomes ranged from 31 to 170. The genome sizes ranged from 4.25 to 6.24 Mb, with a mean size of 5.49 Mb.

### Phylogenetic and functional properties of *Aquimarina* genomes

**Fig. 1:**
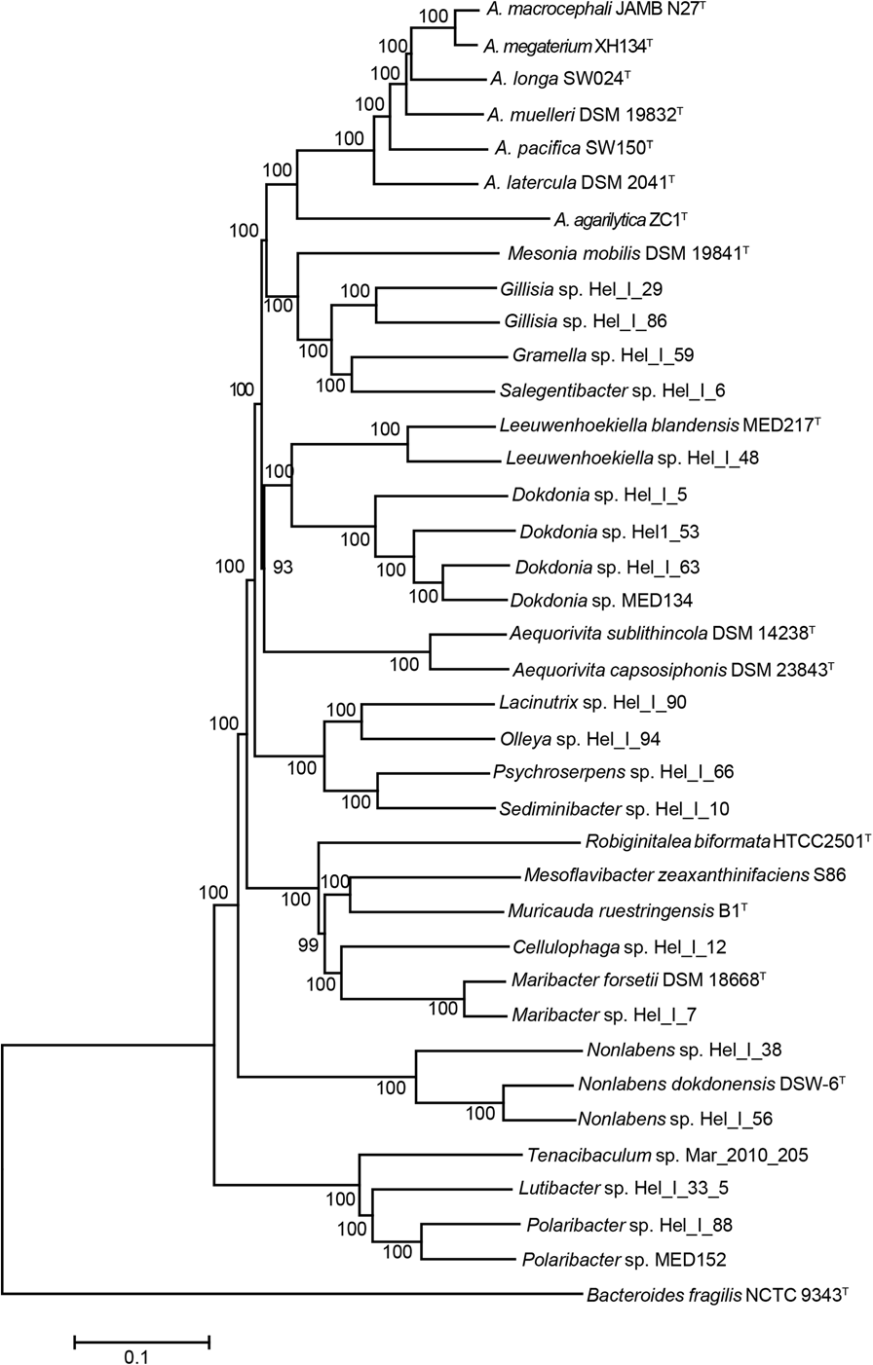
Phylogenetic relationships of the family *Flavobacteriaceae*. The tree was constructed with concatenated alignment of orthologous proteins using Neighbour-joining method with 1000 bootstrap replications. Type species *Bacteroides fragilis* NCTC 9343^T^ from *Bacteroidaceae* served as outgroup

Although it is more popular to use 16S rRNA gene to explain phylogenetic relationship of bacteria, phylogenetic tree constructed using orthologous proteins appears to be more accurate to elucidate the genetic relationship among different microbes [32]. In addition to all the seven *Aquimarina* genomes, genomic sequences of 30 other *Flavobacteriaceae* strains isolated from seawater were also obtained to construct an orthologous proteins tree (Fig. 1, Additional file 1). As expected, genomes of the same genus gathered together, and the genera *Mesonia*, *Gillisia*, *Gramella* and *Salegentibacter* formed a clade which was closely related with the genus *Aquimarina*. Although there is diversity among *Flavobacteriaceae*, the adaptation to the degradation of polymeric substances seems to be a common theme [1, 2].

**Fig. 2:**
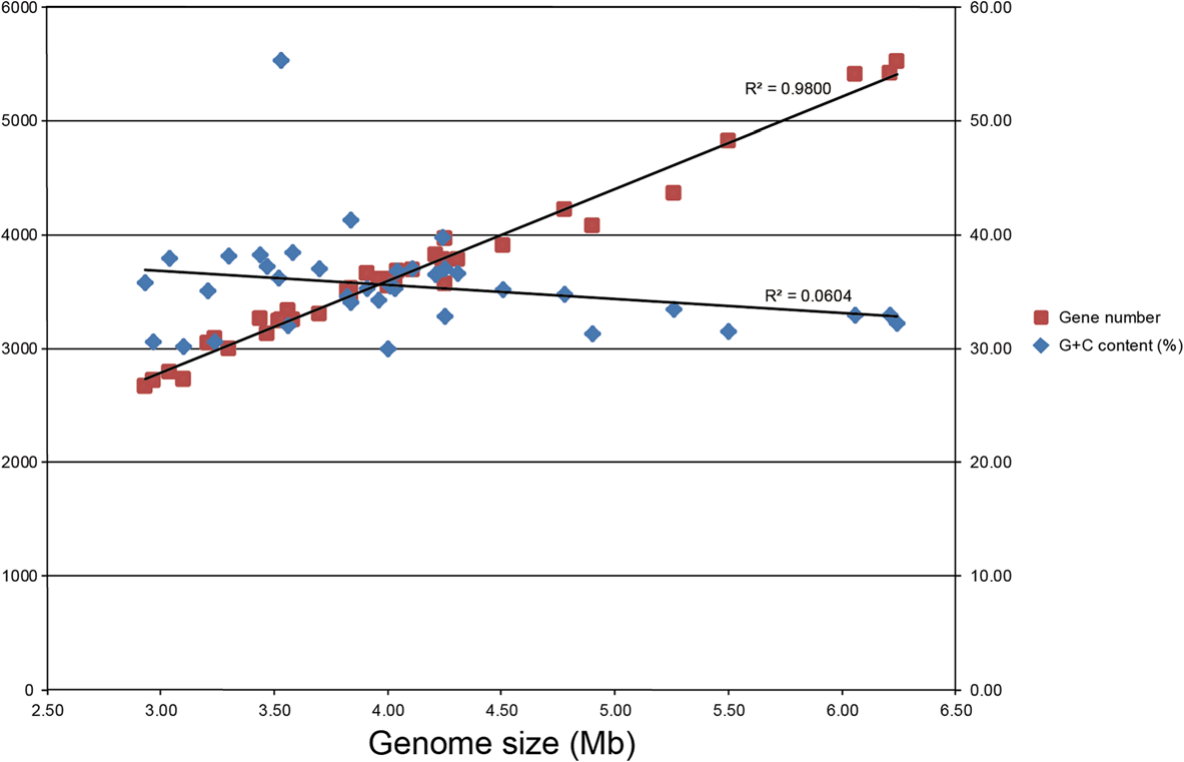
Relationships of genome sizes, ORF numbers and DNA G + C contents. Data of seven *Aquimarina* strains and 30 other *Flavobacteriaceae* strains isolated from seawater were chosen

Analysis of the seven *Aquimarina* genomes and 30 other genomes of *Flavobacteriaceae* isolated from seawater [Additional file 1] showed that ORF numbers were proportional to their genome sizes, with the larger genomes containing greater number of ORFs. The DNA G + C contents of most of these strains were relatively low, ranging from 30 % to 40 % (Fig. 2). Low G + C content may be an adaptive strategy for bacteria to nitrogen limitation [33], because AT base pairs use less nitrogen than GC pairs. The relative availability and/or energetic expenditure incurred by different nucleotides is another explanation for the low G + C content, by the reason that GTP and CTP are energetically more costly to generate than ATP and UTP. Therefore, low G + C content in this genus may help bacteria save energy and would be favored over their GC-rich counterparts when living in the nutrient limited or energetic constraint marine environments.

**Fig. 3:**
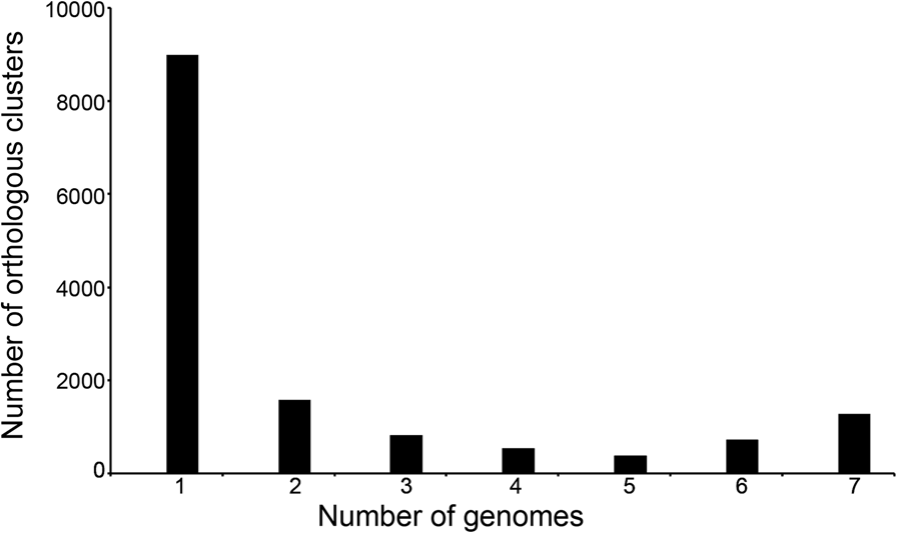
Numbers of orthologous gene clusters that are shared in a given number of *Aquimarina* genomes. One and seven genomes correspond to the unique and core gene clusters, respectively

Meanwhile, orthologous clusters among the seven *Aquimarina* genomes were also analyzed. The number of orthologous clusters contained in each individual genome ranged from 3451 to 5211 (Table 1). Among the strains, *A. muelleri* DSM 19832^T^ had the smallest number of specific genes (869), while *A. latercula* DSM 2041^T^ had the largest (1831). Sixty-three percent (8976) of the total clusters (14,249) were specific genes (Fig. 3), and the proportion was higher than that reported in other genera, such as *Glaciecola* (59 %), *Shewanella* (48 %) and *Streptococcus* (18 %) [32, 34, 35]. In addition, the percentage of core genes (1268, approximately 8.9 %) was lower than that of the specific genes (Fig. 3). These results indicated a high degree of gene content variation in *Aquimarina* genomes, which may be due to the geographic segregation of these strains. Strains of *Aquimarina* in different geographic locations would be unable to exchange genetic information. Instead, they may exchange DNA among surrounding bacteria, thus leading to the high genetic diversity.

The power law and exponential decaying models were used to describe the pan- and core-genome of the genus *Aquimarina*, respectively. The pan-genome curve can perfectly fit a power law function with an exponent of 0.62, which indicates that the pan-genome of *Aquimarina* is open and new orthologous clusters will be added when a new genome of *Aquimarina* is sequenced [36]. The core genome decreased sharply from an average of 4532 to 2300 when the first two genomes were added [Additional file 2].

**Fig. 4:**
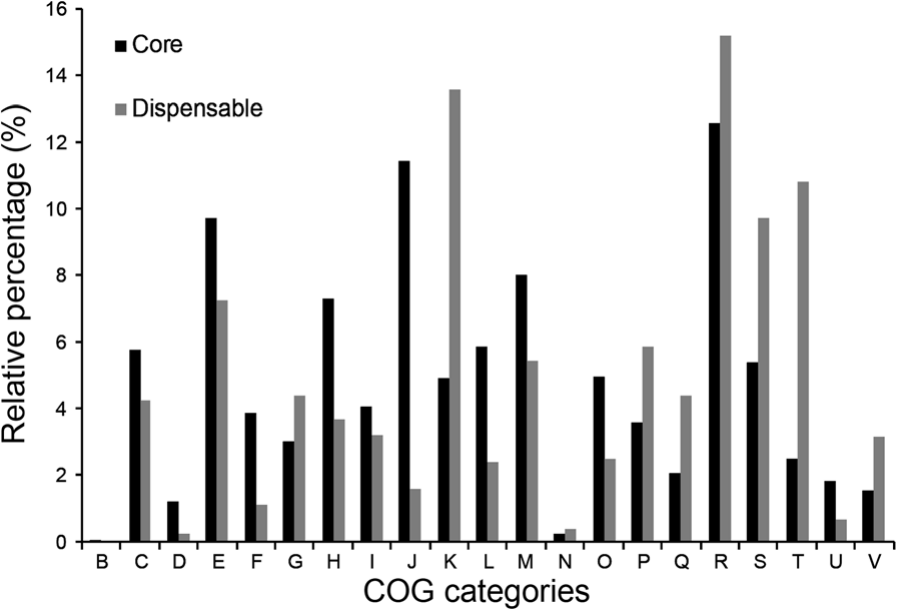
Comparison of the COG categories of the core and dispensable gene sets coding proteins. The function of COG categories is described in Table 2

Functional characterization of COG (Table 2) showed that *Aquimarina* had higher proportions of genes for translation, ribosomal structure and biogenesis (J), signal transduction mechanisms (T), and secondary metabolites biosynthesis, transport and catabolism (Q) compared to the mean values of 115 genomes calculated by Konstantinidis and Tiedje [37]. The *Aquimarina* core gene sets were enriched in genes that encode proteins involved in translation, posttranslational modification, as well as metabolism of amino acid, nucleotide, coenzyme and lipid (COG categories J, O, E, F, H and I) when compared with dispensable genes (Fig. 4). These genes are retained in all the genomes since they are related to central metabolisms and are essential to survival. Similar enrichment was also observed in the genera *Glaciecola* and *Shewanella* [32, 34]. However, the lower proportions of core gene sets corresponding to transcription (K) and signal transduction mechanisms (T) indicate that different genomes may have evolved different transcriptional and signal transduction systems, or alternatively the transcriptional and signaling genes are not well conserved in this genus.

**Table 2:** Percentage of COG categories in each *Aquimarina* strain

COG categories	*A. longa* SW024^T^	*A. pacifica* SW150^T^	*A. megaterium* XH134^T^	*A. macrocephali* JAMB N27^T^	*A. latercula* DSM 2041^T^	*A. muelleri* DSM 19832^T^	*A. agarilytica* ZC1^T^
B	0.036	0.038	0.029	0.030	0.031	0.037	0.046
C	4.753	4.641	4.548	4.519	4.734	4.978	5.251
D	0.756	0.761	0.583	0.667	0.611	0.780	0.883
E	7.886	7.607	8.192	8.098	7.086	8.841	7.574
F	2.233	2.396	2.012	2.032	2.046	2.489	2.463
G	3.277	4.108	4.052	3.518	5.559	3.306	5.112
H	5.041	4.869	4.548	4.580	4.276	5.163	4.879
I	3.169	3.119	3.528	3.397	3.787	3.975	3.253
J	5.906	6.162	4.956	5.126	5.162	6.241	7.342
K	8.246	8.406	11.079	10.889	8.888	7.578	6.831
L	6.158	4.336	3.236	4.246	3.940	4.755	5.762
M	7.742	8.939	6.239	6.127	8.125	7.355	9.201
N	0.360	0.380	0.262	0.303	0.458	0.371	0.743
O	3.745	3.728	3.178	3.518	3.635	4.123	4.507
P	5.149	5.401	4.519	4.610	4.795	4.866	5.390
Q	2.953	3.081	3.761	3.518	3.451	4.383	2.138
R	13.936	13.998	14.519	14.680	14.508	14.042	13.151
S	8.714	7.950	7.872	8.432	8.522	7.912	7.667
T	6.482	6.618	8.542	7.583	6.659	5.498	4.926
U	1.296	1.445	1.137	1.092	1.100	1.226	1.115
V	2.161	2.016	3.178	3.033	2.627	2.043	1.766
W	0.000	0.000	0.029	0.000	0.000	0.037	0.000

Function of COG categories: [B] Chromatin Structure and dynamics; [C] Energy production and conversion; [D] Cell cycle control, cell division, chromosome partitioning; [E] Amino acid transport and metabolism; [F] Nucleotide transport and metabolism; [G] Carbohydrate transport and metabolism; [H] Coenzyme transport and metabolism; [I] Lipid transport and metabolism; [J] Translation, ribosomal structure and biogenesis; [K] Transcription; [L] Replication, recombination and repair; [M] Cell wall/membrane/envelope biogenesis; [N] Cell motility; [O] Posttranslational modification, protein turnover, chaperones; [P] Inorganic ion transport and metabolism; [Q] Secondary metabolites biosynthesis, transport and catabolism; [R] General function prediction only; [S] Function unknown; [T] Signal transduction mechanisms; [U] Intracellular trafficking, secretion, and vesicular transport; [V] Defense mechanisms; [W] Extracellular structures

### Comparison of three *Aquimarina* strains isolated from SPG

**Fig. 5:**
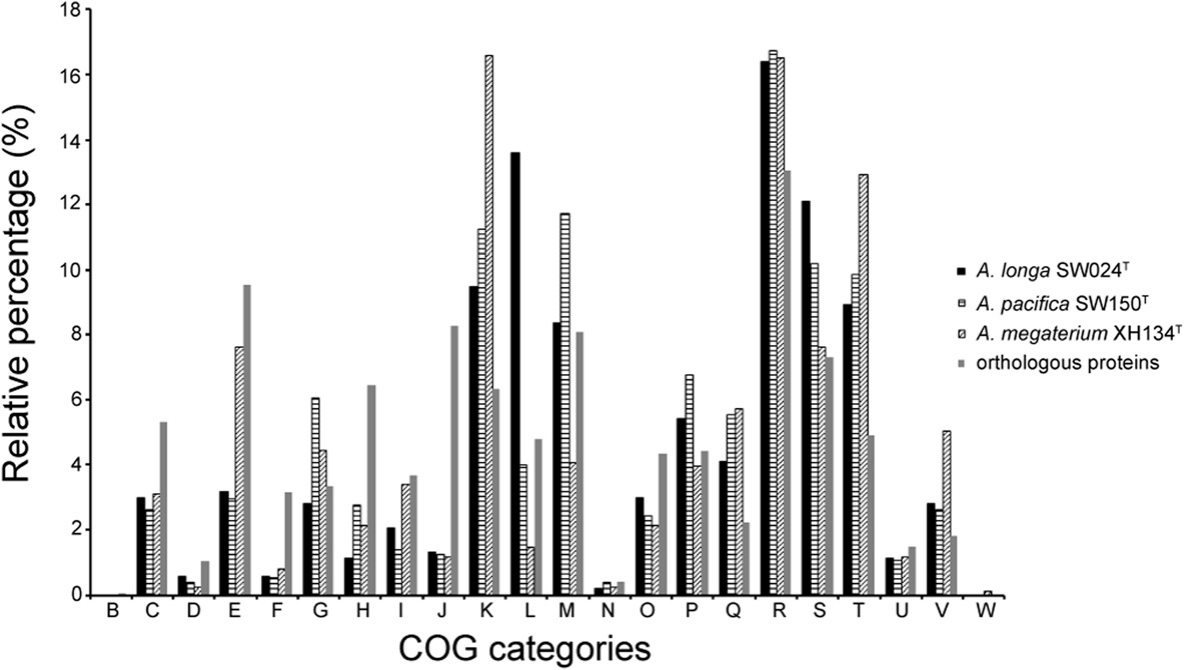
Relative abundance compared to all COG categories of the orthologous and specific proteins. Putative orthologous proteins are defined as reciprocal best hit proteins with a minimum 50 % identity and 70 % of the length of the query protein, calculated by the BLAST algorithm. The function of COG categories is described in Table 2

*A. longa* SW024^T^ [10], *A. pacifica* SW150^T^ [21] and *A. megaterium* XH134^T^ [20] were isolated from surface seawater of the SPG at stations U1367, U1369 and U1371, respectively, as shown in [11]. The COG-based analysis among the specific and orthologous proteins showed that the largest proportion of orthologous genes of the three bacteria belong to amino acid transport and metabolism (E), while that of specific genes in *A. longa* SW024^T^, *A. pacifica* SW150^T^ and *A. megaterium* XH134^T^ belong to replication, recombination and repair (L), cell wall/membrane/envelope biogenesis (M) and transcription (K), respectively (Fig. 5). The different genome contents among these bacteria may be likewise correlated with diverse phylogenies, trophic strategies and ocean environments, as described in roseobacters [38]. For instance, *A. longa* SW024^T^ possesses four photolyases PhrB, while three and only one PhrB coding genes were identified in *A. pacifica* SW150^T^ and *A. megaterium* XH134^T^, respectively. This might be one of the reasons to explain their diverse distributions. With the lowest Chl *a* in station U1367, bacteria in this place suffer more UV damage than those in other stations and therefore needs more PhrB.

### Bacterial shape and growth of *A. longa* SW024^T^

**Fig. 6:**
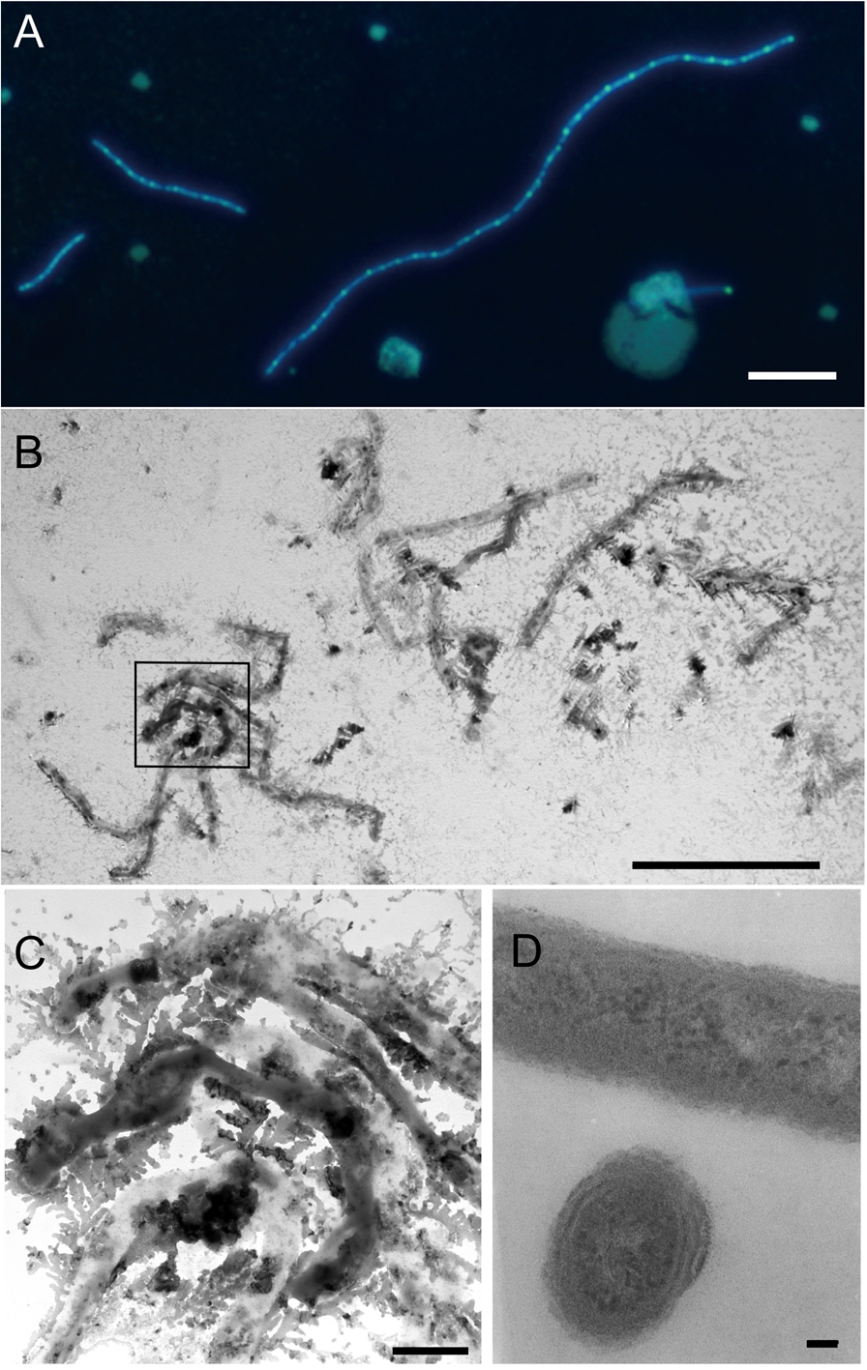
Bacterial shape of *A. longa* SW024^T^. Fluorescence microscopy of *A. longa* SW024^T^ stained with DAPI, bar = 5 μm (**a**). Transmission electron microscopy of *A. longa* SW024^T^ culturing in MB medium without staining, bar = 10 μm (**b**) and magnification of aggregate section in the boxed area, bar = 1 μm (**c**). Transmission electron microscopy of *A. longa* SW024^T^ using ultramicrotomy, including transections and longitudinal section, bar = 50 nm (**d**)

Cells of *A. longa* SW024^T^ usually appear as filamentous and are relatively long in length (Fig. 6a), the maximum length grown in MB is 66 μm [10], about 100 times longer than the smallest bacteria observed in oligotrophic ocean environment [39]. Moreover, it is thin and the width is only about 0.3 μm (Fig. 6d). *A. megaterium* XH134^T^, which was also isolated from SPG, is even longer (up to 77.8 μm) and harbors a larger genome (6.21 Mb, Table 1). Large genome brings large nucleic acids and proteins, therefore the cell must have sufficient room to include all the nucleic acids, proteins, molecular complexes and other gears required for survival and proliferation [40]. The relatively long lengths might confer an advantage for the bacteria attaching to particle surfaces by increasing the contact area. Proteins functioning in cell size control by maintaining cell shape within normal ranges were found in the genome of *A. longa* SW024^T^, such as FtsZ, penicillin binding protein 2 (PBP 2) and MreBCD.

Growth was measured in a series of diluted MB and MA media to examine its survival in nutrient-limiting conditions. The result showed that *A. longa* SW024^T^ could grow on 1:20 dilution of MA plate, but only in 1:2 dilution of MB liquid medium, after two weeks’ culture. *A. longa* SW024^T^ formed yellow colonies on agar plates [Additional file 3] and tended to aggregate into flocks in liquid culture (Fig. 6b). Abundant extracellular materials were secreted which may help bacteria gather together (Fig. 6c). The genome owns a large array of genes involved in the synthesis and export of extracellular polysaccharide material (*e.g.*, 33 putative glycosyl transferases). These results suggest that *A. longa* SW024^T^ may grow in the nutrient-limiting environment by attaching to surfaces of particles.

After being precipitated with ethanol and lyophilized, 0.8 mg mL^−1^ EPS was obtained from *A. longa* SW024^T^ cultured in MB medium. This amount is equal to the production of *Herbaspirillum seropedicae* [30], but less than that of *Pseudomonas atlantic* (mean production is 2.7 mg mL^−1^) [41]. In natural aquatic environments, nutrients required to support maximal microbial growth are rarely present in sufficient quantities in the water column. Microbial attachment and aggregates is likely a strategy to increase the rate of substrate uptake [42], and a porous matrix of exopolymer surrounding microbial cells could sequester and concentrate dissolved organic compounds [41]. It thus could be inferred that EPSs synthesized by *A. longa* SW024^T^ might act as a sponge to trap and concentrate nutrients in flowing liquids and be able to help this bacterium absorb dissolved organic material.

Smaller genomes, fewer gene duplications, and depleted in DNA G + C contents, noncoding nucleotides, and genes encoding transcription, signal transduction and noncytoplasmic proteins have been identified as indicators of genome streamlining and oligotrophy [43, 44], all of which are opponent to *A. longa* SW024^T^ except the low DNA G + C content. Genome of *A. longa* SW024^T^ is overrepresented in dehydrogenases (77 predicted dehydrogenases), and is enriched in COGs involved in defense mechanisms (V), transcription (K) and signal transduction (T, Table 2), consisting with the properties of copiotrophs proposed by Lauro et al. [44]. With these genomic and phenotypic features, we assume that *A. longa* SW024^T^ is a copiotroph but can survive in oligotrophic marine environment probably by attaching to particles.

### Gliding motility

*A. longa* SW024^T^ has a complete set of genes involved in gliding motility (*gldA*, *gldB*, *gldC*, *gldD*, *gldE*, *gldF*, *gldG*, *gldH*, *gldI*, *gldJ*, *gldK*, *gldL*, *gldM*, *gldN*, *sprA* and *sprE*), which could be beneficial in the exploration of solid surfaces. *A. longa* SW024^T^ attaches readily to glass slide and displays rapid motility [10]. Previous studies showed that cells with mutations in genes encoding these proteins were completely nonmotile in *Flavobacterium johnsoniae* ([45] and references therein). Mutants did not exhibit movement on agar or glass surfaces, failed to propel latex spheres, and formed nonspreading colonies. The gliding motility of *Aquimarina* may be stimulated by the oligotrophic environment, since it is beneficial to search insoluble macromolecular substrates such as starch and chitin, which could be utilized by *Aquimarina* as carbon resource. Moreover, gliding motility can help bacteria position themselves at optimal conditions of light intensity, oxygen, hydrogen sulfide, temperature and other factors that influence growth [46].

### Adaptation strategies to the oligotrophic marine environment

Survival of a bacterium in the ultra-oligotrophic surface seawater depends on effective uptake of the primary elemental ingredients for life, such as nitrogen (N), phosphorus (P), sulfur (S) and iron (Fe). Many proteins produced by *A. longa* SW024^T^ are involved in these processes (Table 3).

**Table 3:** Genes related to oligotrophic marine environment adaption

Function	Encoded gene product	Gene
Nitrogen sensing and regulation	nitrogen regulation protein	*ntrY*
	nitrogen regulatory protein	*glnB*
Ammonium assimilation	glutamine synthetase	*glnA*
	glutamate synthase, NADH/NADPH small subunit	*gltD*
	glutamate synthase, NADPH/NADH large subnit	*gltB*
Denitrification	nitrate reductase	*napA*
	nitrite reductase	*nirK*
	nitric oxide reductase	*norB*
	nitrous oxide reductase	*nosZ*
Hydrolysis of dissolved organic phosphorus	alkaline phosphatase	*phoD*
Sensing and responding to changes in external/internal P levels	PhoP family transcriptional regulator	*phoP*
	histidine kinase	*phoR*
Sulfate transport	putative sulfate transporter	*cysZ*
	sulfate permease	*sulP*
Iron uptake	TonB-dependent siderophore receptor	/
	iron(III) ABC transporter	/
	ferrous iron transport protein B	*feoB*
	ferrous iron transport protein A	*feoA*
	ferric enterobactin receptor	/
Primary Na + pump	Na^+^-translocating NADH/ubiquinone oxidoreductase subunit A	*nqrA*
	Na^+^-translocating NADH/ubiquinone oxidoreductase subunit B	*nqrB*
	Na^+^-translocating NADH/ubiquinone oxidoreductase subunit C	*nqrC*
	Na^+^-translocating NADH/ubiquinone oxidoreductase subunit D	*nqrD*
	Na^+^-translocating NADH/ubiquinone oxidoreductase subunit E	*nqrE*
	Na^+^-translocating NADH/ubiquinone oxidoreductase subunit F	*nqrF*
Primary H^+^ pump	cytochrome d ubiquinol oxidase subunit I	*cydA*
	cytochrome d ubiquinol oxidase subunit II	*cydB*
	cytochrome c oxidase subunit I	*coxA*
	cytochrome c oxidase subunit II	*coxB*
	cytochrome c oxidase subunit III	*coxC*
	cytochrome c oxidase subunit IV	*coxD*
	protoheme IX farnesyltransferase	*cyoE*
Exporting of Na^+^ ions	Na^+^/H^+^ antiporter	*nhaA*
	Na^+^/H^+^ antiporter	*nhaB*
	Na^+^/H^+^ antiporter	*nhaC*
	Na^+^/H^+^ antiporter	*nhaP*
Other sodium dependent	Na^+^/proline symporter	/
	Na^+^/phosphate symporter	/
	cation/acetate symporter	*actP*
	Na+/dicarboxylate symporter	/
	Na+/nucleoside permease	/
	Na^+^/iodide cotransporter	/
	Na^+^/K^+^/Ca^2+^ exchanger	*yrbG*
	Na^+^/bile acid transporter	/
	Na^+^/multivitamin transporter	/
	Na^+^/glucose cotransporter	/
	proton/Na^+^-glutamate symporter	/
Extracellular osmolarity and salinity stress adaption	MIP family channel protein aquaporin	*aqpZ*

*A. longa* SW024^T^ harbors nitrogen regulation proteins NtrY and P_II_ that are required for sensing and responding to N fluctuation in seawater. The P_II_ signal transduction protein has a central position in the coordination of carbon, nitrogen and energy status of the cells. Most of the target proteins interacting with P_II_ protein perform or regulate crucial reactions in nitrogen assimilatory pathways [47]. In addition, ammonium assimilation in this bacterium is mediated via glutamine synthetase and glutamate synthase. Nitrate is the most abundant N species in ocean environments, a complete pathway of denitrification exists in *A. longa* SW024^T^, helping the bacterium acquire energy by this process.

P starvation may limit growth, and potentially constrain nitrogen fixation. Four genes in the genome are involved in P acquisition, and all of them encode putative alkaline phosphatases, which are necessary for hydrolysis of dissolved organic phosphorus [48]. Two-component system PhoR/PhoP in the genome may play a role in sensing and responding to changes in external/internal P levels prior to activating components of the P acquisition tool kit.

Although the classical ABC-type sulfate transport system is missing in *A. longa* SW024^T^, it encodes two proteins, *i.e.*, a homolog of CysZ and a putative sulfate permease, both of which could serve as a sulfate transporter. Iron uptake mechanisms include TonB-dependent siderophore receptor, ABC-type iron transporter, ferrous iron transport protein, and ferric enterobactin receptor.

Like other marine bacteria, *A. longa* SW024^T^ encodes a primary Na^+^ pump, the Na^+^-translocating NADH/ubiquinone oxidoreductase, and probably uses a sodium ion gradient as the source of energy for nutrient uptake. In addition, it encodes primary H^+^ pumps, namely, cytochrome bd complex, and cytochrome c oxidase. Salt acclimation includes several ion transporters which serve as exporters for sodium and chloride, the main toxic ions in seawater, and importers for potassium, which is essential for many cellular processes. Exporting of Na^+^ ions at the expense of the proton gradient is performed by a variety of Na^+^/H^+^ antiporters, including NhaA, NhaB, NhaC and NhaP. In addition, *A. longa* SW024^T^ harbors Na^+^/proline symporter, Na^+^/phosphate symporter, cation/acetate symporter, Na^+^/dicarboxylate symporter, Na^+^/nucleoside permease, Na^+^/iodide cotransporter, Na^+^/K^+^/Ca^2+^ exchanger, Na^+^/bile acid transporter, Na^+^/multivitamin transporter, Na^+^/glucose cotransporter, and proton/Na^+^-glutamate symporter. Besides, three aquaporins AqpZ could help the bacterium to withstand dramatic changes in extracellular osmolarity and adapt to salinity stress [49].

### Resistance to adverse effects

Oxidative DNA damage is a major source of mutation load in living organisms by means of damaging DNA, proteins and membranes of cells [50]. To avoid oxidative damage, *A. longa* SW024^T^ has set up several antioxidant defense mechanisms comprising antioxidant enzymes as well as antioxidative compounds. Three types of superoxide dismutase (SOD, *i.e.*, Cu-Zn SOD, Mn-SOD, Fe-SOD) which catalyze the dismutation of O_2_^−^ to O_2_ and H_2_O_2_, have been identified in the *A. longa* SW024^T^ genome. Genes encoding for two catalase-peroxidases, KatG and KatE, catalyzing the decomposition of hydrogen peroxide to water and oxygen are present in the genome. Moreover, it was reported that translation of the quinone-binding proteins was protected by the *katE* gene in tobacco leaves during exposure to light stress [51]. These antioxidants might also be crucial for bacteria survival during exposure to other stresses such as UV radiation. The reactive oxygen species-scavenging system in *A. longa* SW024^T^ also contains three peroxiredoxins (Prx), termed thioredoxin peroxidases, including one PrxQ and two 2-Cys Prx, which catalyze the reduction of various hydroxyperoxides. Prx proteins mainly function when the concentration of H_2_O_2_ is low, while catalases mainly detoxify high H_2_O_2_ levels [52], although both of them decompose H_2_O_2_. Several genes encoding thioredoxins (Trx) and Trx-like proteins are also found in the genome, including TrxA and TrxB. In addition, *A. longa* SW024^T^ possesses gene encoding for MutY which may prevent mutations arising from oxidatively damaged guanine residues [53].

Inhabiting in such clear surface seawater, *A. longa* SW024^T^ has evolved several genetic potentials for UV radiation defense. Corresponding genes involved in the biosynthetic pathways of C_40_ carotenoids (*i.e.*, *crtE*, *crtB*, *crtI*, *crtY*, *crtZ*) could be identified in the genome of *A. longa* SW024^T^. In addition to protecting cells against damaging radicals resulting from the degradation of heterocycles [54], carotenoids can also function in resisting to photodestruction [55]. Hence, the synthesis of carotenoids may protect *A. longa* SW024^T^ from UV damage in the clear seawater. Moreover, *A. longa* SW024^T^ possesses four putative photolyases PhrB, which are DNA repairing flavoproteins that response to blue light and repair cyclobutane pyrimidine (mainly thymine) dimers created by UV light. One BLUF domain protein which has been shown to sense blue light is found in the genome. Similar domain was also found in other marine *Flavobacteriaceae* bacteira [2]. In addition, genes involved in nucleotide excision repair (NER) are also found in *A. longa* SW024^T^, including *uvrA*, *uvrB*, *uvrC* and *uvrD* [56]. The NER system has an advantage over photolyase in that it can repair UV lesions in the dark. Moreover, the key protein in the transcription coupled repair process is a transcription repair coupling factor (TRCF) encoded by the *mfd* gene [57]. Mfd recognizes RNA polymerase stalled at a non-coding template site of DNA damage, disrupts the transcription complex to release the transcript and enzyme, and binds UvrA via the UvrA-binding domain 2 that is very similar to domain 2 of UvrB thereby recruiting the NER machinery to the DNA lesion [56]. Further, three 6-O-methylguanine-DNA methyltransferases can also help repair alkylated forms of guanine and thymine that can lead to G:C to A:T transversions in DNA [58].

The *in vitro* antibiotic sensitivity test demonstrated multidrug resistance pattern of *A. longa* SW024^T^, with resistance to 12 antibiotics [10]. A variety of known antibiotic-resistance proteins, such as *β*-lactamase (AmpC), outer membrane proteins (OmpA, OmpW), and potential drug transporters were found in the genome of *A. longa* SW024^T^. The bacterium is resistant to *β*-lactam antibiotics, *i.e.*, benzylpenicillin, carbenicillin, cefuroxime and cephalosporin. Several *β*-lactamases encoding genes were identified in the genome. Thus, the inactivation of the antibiotics via degradation by *β*-lactamases seems to be an intrinsic resistance mechanism. It is also resistant to aminoglycosides, *i.e.*, gentamicin, kanamycin, neomycin and streptomycin. Multidrug efflux pumps also play important roles in *A. longa* SW024^T^ antimicrobial resistance. A large number of drug transporters and efflux pumps were identified in the genome, including multidrug ABC transporter, SMR family multidrug resistance protein, cation/multidrug efflux pump, ABC efflux pump, Na^+^ driven efflux pump and MATE efflux pump. These multidrug transporters recognize lipophilic drugs by their physic-chemical properties that allow them to intercalate into the lipid bilayer, and transport these agents from the lipid bilayer to the exterior. Antimicrobial activity is of help for *A. longa* SW024^T^ to compete with opponents in the same environment for survival, and might also help the bacterium use antibiotics-like substances as energy source and adapt to the ultra-oligotrophic marine environment.

### Potential role in biogeochemical cycles

Chitin is the most abundant renewable biopolymers in the marine environment. It has been estimated that 10^11^ tons of chitin are produced annually in marine systems, primarily in the form of zooplankton exoskeletons, and this polymer must be continually remineralized to support sustained primary production in the oceans [59]. Thus, degradation of chitin may reflect one of the most important extracellular enzymatic processes in the marine environment and create important trophic links within bacterioplankton communities. From the chitinase activity assay, chitin can be hydrolyzed by *A. longa* SW024^T^ [Additional file 3]. Chitinase is a glycosyl hydrolase which catalyzes the degradation of chitin. Based on amino acid sequence similarity, chitinases are classified into families 18 and 19 of glycosyl hydrolases [60, 61]. *A. longa* SW024^T^ harbored seven genes encoding chitinase, four of which belong to family 19 (blast using UNIPROTKB database), which is an interesting result because most of the family 19 chitinases were found in higher plants. In recent years, genus *Aquimarina* was isolated in many oceanic areas, and it may play a role in the cycling of nutrients especially for carbon in the oceans.

Denitrification constitutes one of the main branches of the global nitrogen cycle sustained by bacteria. For nitrogen metabolism, a complete pathway of denitrification was found in the genome of *A. longa* SW024^T^, which is the process of converting nitrate (NO_3_^−^) to nitrite (NO_2_^−^), nitric oxide (NO), nitrous oxide (N_2_O) and dinitrogen gas (N_2_), making use of N oxides as terminal electron acceptors for cellular bioenergetics. Moreover, genes involved in the pathway of assimilatory sulfate reduction were also found in the genome, which converts sulfate to sulfide. The ability to consume a wide array of carbon, nitrogen and sulfur substrates indicates that the bacterium might play an important role in biogeochemical cycles.

## Conclusions

Genome comparison of *Aquimarina* strains suggest that the genome contents of these bacteria are in line with their living environments. The general features of *A. longa* SW024^T^ genome are consistent with its life style in the surface ocean. With large genome size, a large number of ORFs and COG categories comparable to other copiotrophs, *A. longa* SW024^T^ is assumed to be a copiotroph. Living in the ultra-oligotrophic marine environment, *A. longa* SW024^T^ is more likely abundant on particles than free-living in the water column, and search for polymers by its gliding ability. A series of adaptation strategies to oligotrophic marine environment including uptake of the primary elemental ingredients such as N, P, S and Fe were identified in the genome. Antioxidative enzymes and compounds as well as other antibiotic activity proteins here might help the bacterium resistant to adverse effects such as DNA damage. Carbon, nitrogen and sulfate metabolism indicate that the bacterium may play a role in biogeochemistry cycle. The analysis of the genome of *A. longa* SW024^T^ presented here provides a better understanding of its survival mechanisms and ecophysiological functions in the ultra-oligotrophic marine environment.

## Availability of supporting data

The data sets supporting the results of this article are included within the article.

## Additional files

Additional file 1:
**Summary of genomic information of 30** ***Flavobacteriaceae*** **genomes.** (DOCX 21 kb) Additional file 2:
**Pan-genome and Core-genome of the genus** ***Aquimarina*** **.** Squares are the values obtained for the different strain combinations of Pan-genome. Circles are the values obtained for the different strain combinations of Core-genome. Triangles are the average of such values. The curves are the least squares fit of the power law to the average values. (TIFF 410 kb) Additional file 3:
**Chitinase activity of** ***A.longa*** **SW024** ^**T**^ **.** (TIFF 1703 kb)

## Abbreviations

BD: Becton dickinson
BLAST: Basic local alignment search tool
Chl a: Chlorophyll a
COG: Clusters of orthologous groups of proteins
DDBJ: DNA data bank of japan
EMBL: European molecular biology laboratory
EPS: Exopolysaccharides
GO: Gene ontology
GOLD: Genomes online database
KEGG: Kyoto encyclopedia of genes and genomes
MA: Marine Agar 2216
MB: Marine Broth 2216
NCBI: National center for biotechnology information
NER: Nucleotide excision repair
NR: Non-Redundant
ORF: Open reading frame
PBP 2: Penicillin binding protein 2
Prx: Peroxiredoxin
SOD: Superoxide dismutase
SPG: South Pacific Gyre
TRCF: Transcription repair coupling factor
Trx: Thioredoxin
