# Supplementary material for: Genomic insight into Aquimarina longa SW024T: its ultra-oligotrophic adapting mechanisms and biogeochemical functions
Source: BMC Genomics. 2015 Oct 12;16:772. doi: 10.1186/s12864-015-2005-3 (PMC4603819; doi:10.1186/s12864-015-2005-3)
Supplement: Additional file 1: — Summary of genomic information of 30 Flavobacteriaceae genomes. (DOCX 21 kb) [file 12864_2015_2005_MOESM1_ESM.docx]

**Additional file 1 Summary of genomic information of 30 *Flavobacteriaceae* genomes.**

| Strains | Size (Mb) | G+C content (%) | ORF number | Bioproject Accession | Isolated environment |
| --- | --- | --- | --- | --- | --- |
| *Aequorivita capsosiphonis* DSM 23843^T^ | 4.04 | 36.84 | 3630 | PRJNA185523 | seawater |
| *Aequorivita sublithincola* DSM 14238^T^ | 3.52 | 36.19 | 3198 | PRJNA46631 | seawater |
| *Cellulophaga* sp. Hel_I_12 | 4.03 | 35.30 | 3508 | PRJNA251768 | seawater |
| *Dokdonia* sp. Hel_I_5 | 3.58 | 38.48 | 3195 | PRJNA262383 | seawater |
| *Dokdonia* sp. Hel1_53 | 2.93 | 35.77 | 2608 | PRJNA265336 | seawater |
| *Dokdonia* sp. Hel_I_63 | 3.47 | 37.26 | 3038 | PRJNA257847 | seawater |
| *Dokdonia* sp. MED134 | 3.30 | 38.16 | 2944 | PRJNA13544 | seawater |
| *Gillisia* sp. Hel_I_29 | 3.96 | 34.30 | 3545 | PRJNA217202 | seawater |
| *Gillisia* sp. Hel_I_86 | 4.25 | 36.98 | 3870 | PRJNA257846 | seawater |
| *Gramella* sp. Hel_I_59 | 3.44 | 38.21 | 3192 | PRJNA257842 | seawater |
| *Lacinutrix* sp. Hel_I _90 | 3.82 | 34.61 | 3447 | PRJNA251769 | seawater |
| *Leeuwenhoekiella blandensis* MED217^T^ | 4.24 | 39.76 | 3735 | PRJNA13573 | seawater |
| *Leeuwenhoekiella* sp. Hel_I_48 | 4.31 | 36.57 | 3680 | PRJNA217201 | seawater |
| *Lutibacter* sp. Hel_I_33_5 | 3.10 | 30.15 | 2670 | PRJNA265337 | seawater |
| *Maribacter* sp. Hel_I _7 | 4.78 | 34.75 | 4151 | PRJNA217216 | seawater |
| *Maribacter forsetii* DSM 18668^T^ | 4.51 | 35.18 | 3843 | PRJNA217215 | seawater |
| *Mesoflavibacter zeaxanthinifaciens* S86 | 3.70 | 37.06 | 3256 | PRJNA65289 | seawater |
| *Mesonia mobilis* DSM 19841^T^ | 3.21 | 35.12 | 2945 | PRJNA185643 | seawater |
| *Muricauda ruestringensis* B1^T^ | 3.84 | 41.36 | 3478 | PRJNA52467 | seawater |
| *Nonlabens dokdonensis* DSW-6^T^ | 3.91 | 35.32 | 3618 | PRJNA33015 | seawater |
| *Nonlabens* sp. Hel_I_38 | 3.04 | 37.99 | 2735 | PRJNA262408 | seawater |
| *Nonlabens* sp. Hel_I_56 | 4.01 | 35.44 | 3551 | PRJNA262416 | seawater |
| *Olleya* sp. Hel_I_94 | 3.56 | 32.00 | 3222 | PRJNA257845 | seawater |

**Additional file 1 Summary of genomic information of 30 *Flavobacteriaceae* genomes (continued).**

| *Polaribacter* sp. Hel_I_88 | 4.00 | 29.99 | 3450 | PRJNA217200 | seawater |
| --- | --- | --- | --- | --- | --- |
| *Polaribacter* sp. MED152 | 2.97 | 30.61 | 2679 | PRJNA13543 | seawater |
| *Psychroserpens* sp. Hel_I_66 | 3.84 | 34.06 | 3416 | PRJNA251763 | seawater |
| *Robiginitalea biformata* HTCC2501^T^ | 3.53 | 55.29 | 3209 | PRJNA13461 | seawater |
| *Salegentibacter* sp. Hel_I_6 | 4.21 | 36.55 | 3708 | PRJNA248520 | seawater |
| *Sediminibacter* sp. Hel_I_10 | 4.11 | 36.99 | 3637 | PRJNA217214 | seawater |
| *Tenacibaculum* sp. Mar_2010_205 | 3.24 | 30.53 | 2976 | PRJNA265356 | seawater |
