# Supplementary figures and images for: Genomic insight into Aquimarina longa SW024T: its ultra-oligotrophic adapting mechanisms and biogeochemical functions
Source: BMC Genomics. 2015 Oct 12;16:772. doi: 10.1186/s12864-015-2005-3 (PMC4603819; doi:10.1186/s12864-015-2005-3)

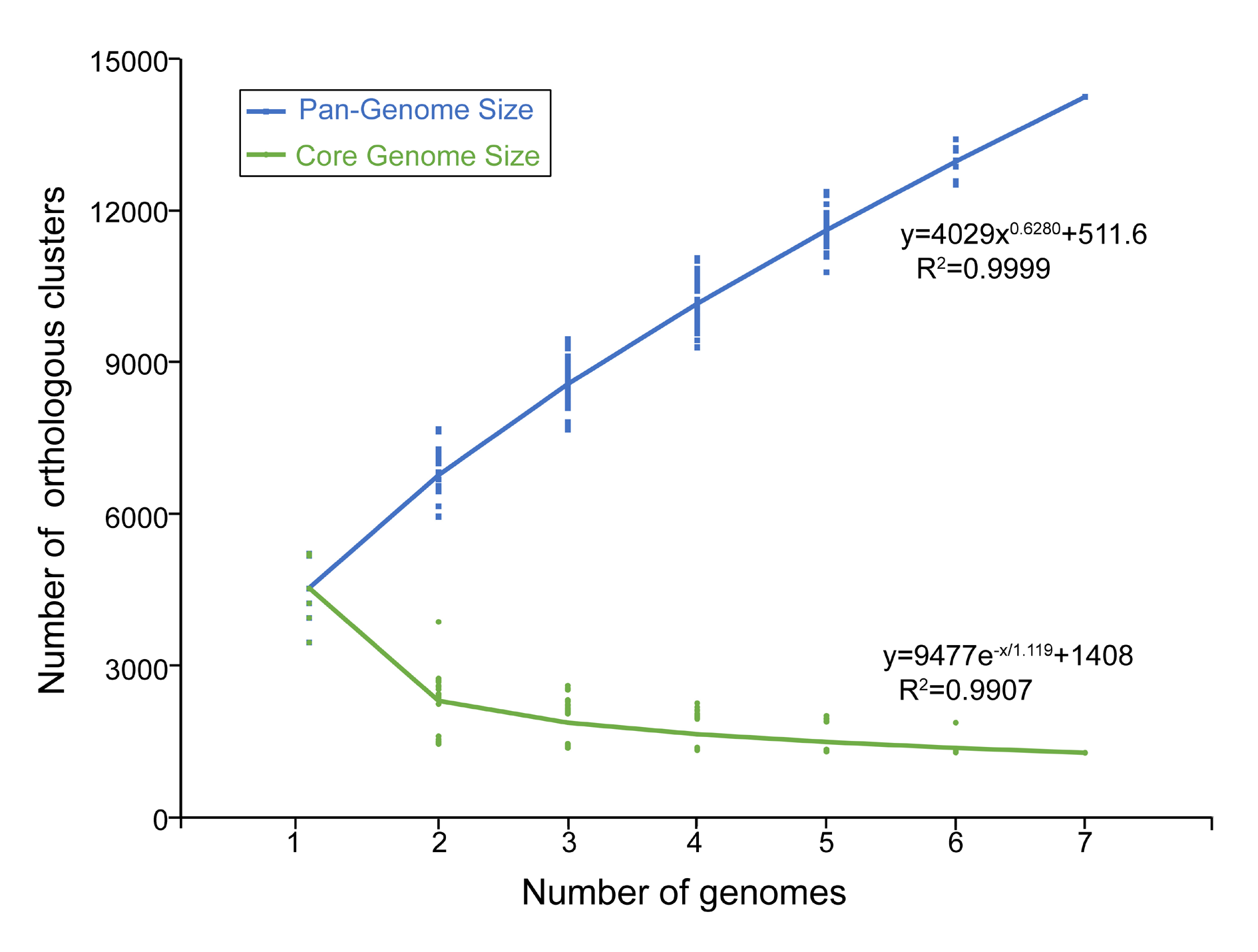

Supplement: Additional file 2: — Pan-genome and Core-genome of the genus Aquimarina . Squares are the values obtained for the different strain combinations of Pan-genome. Circles are the values obtained for the different strain combinations of Core-genome. Triangles are the average of such values. The curves are the least squares fit of the power law to the average values. (TIFF 410 kb) [file 12864_2015_2005_MOESM2_ESM.tif]

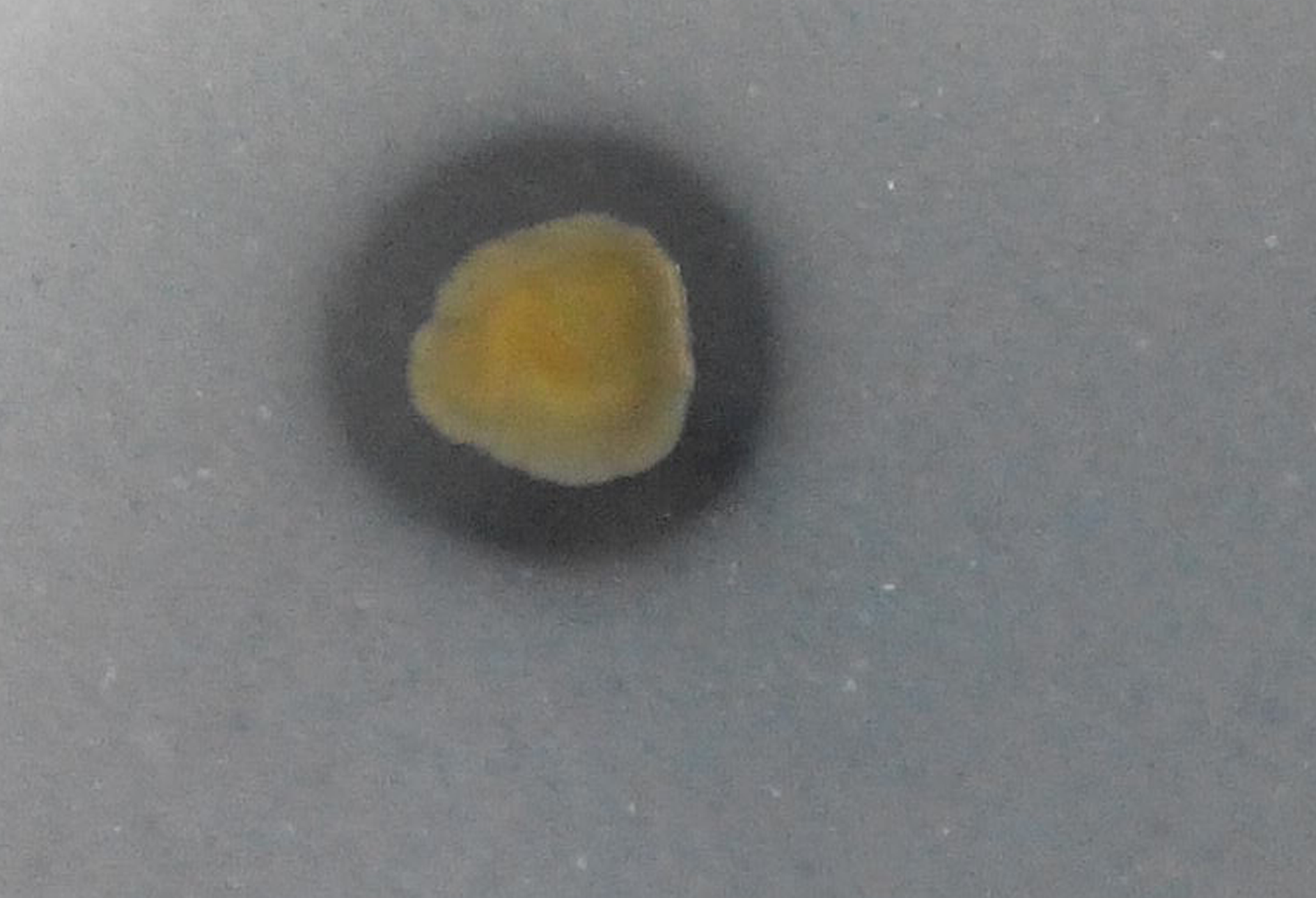

Supplement: Additional file 3: — Chitinase activity of A.longa SW024 T . (TIFF 1703 kb) [file 12864_2015_2005_MOESM3_ESM.tif]
